# Violence experienced by women, their mental health status, and determinants in favelas under the pandemic COVID-19 in Brazil

**DOI:** 10.1186/s12905-025-03793-1

**Published:** 2025-05-21

**Authors:** Mônica Chiodi Toscano de Campos, Rander Junior Rosa, Leticia Perticarrara Ferezin, Thaıs Zamboni Berra, Heriederson Savio Dias Moura, Ariela Fehr Tartaro, Yan Mathias Alves, Reginaldo Bazon Vaz Tavares, Natacha Martins Ribeiro, Juliana Soares Tenório de Araújo, Fernanda Bruzadelli Paulino da Costa, Regina Célia Fiorati, Maria Del Pilar Serrano-Gallardo, Ricardo Alexandre Arcêncio

**Affiliations:** 1 Department of Nursing, Faculty Health, University of Brasilia, Brasilia, Distrito Federal, Brazil; 2 Department of Maternal-Infant Nursing and Public Health, University of São Paulo at Ribeirão Preto College of Nursing, Ribeirão Preto, São Paulo, Brazil; 3 Department of Neurosciences and Behavioral Sciences, Ribeirão Preto Medical School of the University of São Paulo, Ribeirão Preto, São Paulo, Brazil; 4 Autonomous University of Madrid, Madrid, Spain; 5 Instituto de Investigación Sanitaria Puerta de Hierro-Segovia Arana, Majadahonda, Majadahonda Spain; 6 Instituto Interuniversitario de Investigación Avanzada Sobre Evaluación de la Ciencia y la Universidad, Madrid, Spain

**Keywords:** Women, Mental health, Violence, COVID-19, Pandemic

## Abstract

**Background:**

Women in situations of vulnerability experience situations of violence aggravated by their social condition and which generate mental health problems, and these conditions have been further exacerbated by the COVID-19 pandemic.

**Objective:**

This study aims to scrutinize the frequency of violence against women and their mental health status within favelas during the COVID-19 pandemic in Brazil, exploring how the pandemic may have impacted these issues and identifying factors that contribute to these experiences.

**Methods:**

It is a cross-sectional and quantitative study in which data were obtained through the application of a questionnaire adapted and validated for the Brazilian context. The sample consisted of Brazilian women over 18 years old, who lived lived in favelas in the capital cities of Brazilian states. Sampling was carried out using a sequential approach, including participants as they were located and agreed to take part in the study. Descriptive analyses (absolute and relative frequency) were carried out to characterize the profile of the people who answered the questionnaire and logistic regression to identify the factors associated with mental health status and violence against women.

**Results:**

A total of 766 women living in favelas answered the questionnaire. The majority identified themselves as black/brown (75.8%), single, separated, or widowed (51.7%), had completed high school (43.1%), worked informally (47.1%) and did not receive government assistance (57.3%). Black/brown women (OR: 1,05; IC95% 4,55 − 2,53) who were married (OR: 2,45; IC95% 1,18 − 5,19) and exposed to drug trafficking (OR: 3,23; IC95% 1,36 − 7,97) were more likely to experience conjugal violence during the pandemic. In addition, women with informal employment (OR: 3,26; IC95%: 1,24 − 9,55) and complete higher education (OR: 4,47; IC95%: 1,68 − 1,21), were more likely to consider their mental health as precarious during the pandemic period.

**Conclusions:**

The pandemic has exacerbated the social vulnerability of these women, highlighting the need to incorporate a gender perspective into the formulation of public policies during health crises such as the COVID-19 pandemic.

**Supplementary Information:**

The online version contains supplementary material available at 10.1186/s12905-025-03793-1.

## Introduction

The COVID-19 pandemic was declared by the World Health Organization (WHO) on March 11, 2020 [1] and has significantly affected the lives of the general population. To minimize the harmful effects of the COVID-19 pandemic, based on scientific evidence, the WHO recommended that national authorities implement changes in habits in populations, including social distancing, which has become the most effective measure in preventing the spread of the virus. This helps prevent the disease curve from reaching its peak in an accelerated manner, with the risk of overloading health services. However, these recommendations have triggered sudden changes in the lives of families and the population in general, with a negative impact on economic activities, and on all levels of life in society.

In April 2020, the Lancet editorial [2] discussed how the health and social crisis resulting from COVID-19 would disproportionately affect both the rich and poor, with gender inequalities being a determining factor. In all previous humanitarian crises, access to care services for gender-based violence, mental health, and maternal and child health has been reduced [2]. In Brazil, the pandemic arrived at a time when the country was already grappling with significant negative consequences in public policies. Among these challenges was the worsening lack of investment in the Unified Health System (SUS – Sistema Único de Saúde) and the dismantling of public policies for women, particularly those aimed at preventing and addressing gender-based violence.

Despite the serious health situation, the pandemic has shed light on and amplified society’s structural ills [3], including gender-based violence, which is already a serious social problem that has been historically invisible. It is important to consider that women who experience domestic violence accumulate factors of oppression and social stratification. These factors can result from their gender, social class, race and processes of stigmatization due to the intersection of various social markers of difference [4] attributed to them, constituting barriers to seeking help [5].

In Brazil, black women from the favelas, most of whom are heads of household and single mothers, were punished not only for being forced to expose themselves to the virus, but also for exposing the truth of a system in which certain lives are deemed more than others. Not by chance, one of the first victims of COVID-19 in Brazil was a domestic worker [6].

Favelas are characterized by high population density, precarious housing, and insufficient provision of public services, such as water supply and garbage collection, among other precariousness [7], further increasing their situation of risk and vulnerability, especially for those most fragile within a capitalist production system [8].

Finally, Violence against women is a complex phenomenon that manifests in various forms, affecting different groups of women in unequal ways. According to the UN definition, this violence is “any gender-based act that results in, or could result in, physical, sexual, or mental harm, or that involves threats, coercion, or arbitrary deprivation of liberty in public or private life” [9]. Violence against women is a severe human rights violation, widely recognized in international literature [10–12]. Recent studies highlight that violence against women does not affect all groups equally. Physical and sexual violence is often associated with violent behaviors by intimate partners, and its prevalence is alarming, with approximately 30% of women worldwide reporting being victims of physical and/or sexual violence [13]. This phenomenon is exacerbated by gender inequality and social norms that perpetuate men’s power over women, including in domestic and workplace settings. Non-physical violence can be categorized into four types: emotional, psychological, social, and economic violence [14]. It is crucial for women to be aware of the types of violence to which they are exposed and what they should do when they experience violence [15, 16].

However, gender-based violence is often reinforced by inadequate legal systems or a lack of enforcement of existing laws, which prevents true protection for women victims. This can be seen in contexts where the reporting of violence is low due to social, cultural, and legal barriers. Research on violence against women also highlights how lack of access to justice and support services contributes to the perpetuation of the cycle of violence [17]. Gender inequality is a fundamental factor that increases women’s vulnerability to violence, especially for those in situations of extreme poverty, marginalization, or forced displacement. Black, transgender, and migrant women face additional forms of violence, often without adequate access to legal or support resources, further exacerbating their conditions. The intersection of gender, race, and social class is critical in understanding the violence these women face, as demonstrated in research on violence in humanitarian crisis contexts and marginalized populations [17].

The transgenerational nature of this violence is observed in the life history of people in violent relationships [18], and there is a relationship between marital violence and experiences of violence in the family of origin, for both men and women. Intimate partner violence (IPV) impacts the entire family that experiences and witnesses these situations of violence, and they develop physical and emotional problems, such as sleep disorders, anxiety, depression, low self-esteem, and poor school performance, as also indicated by the WHO [19].

During the pandemic, we can highlight some of the situations experienced, such as women who are victims of domestic violence and remain confined with their aggressor, substance abuse, in which women may become alcohol and/or drug users or live as the spouse of a person who is dependent on psychoactive substances and is a trigger for conflict. LGBTQIA + women who have been discriminated against by their own family members experience self-harm, the use of psychoactive substances, and suicidal ideation, with have been exacerbated in this population [20, 21]. Thus, the effects of COVID-19 may be exacerbated for victims of violence, as research shows that well-documented comorbidities of violence include substance abuse and mental health problems. According to one analysis, more than half of women seeking mental health treatment have suffered violence by their partner [22, 23], and it has been associated with depression, post-traumatic stress disorder, anxiety, dysthymia, and phobias [24, 25].

Considering the compelling circumstances elucidated in the literature, primarily attributable to the challenges faced by women in vulnerable territories exacerbated by the pandemic, our objective were to scrutinize the frequency of violence against women, their mental health status of women living in Brazilian favelas during the COVID-19 pandemic. Furthermore, we aimed to investigate the extent to which the pandemic may have influenced these issues, considering social and economic factors that could contribute to increased vulnerability.”

## Methods

### Study design

This is a cross-sectional and quantitative study [26], in which data was collected using a questionnaire adapted and validated for Brazil. The study was carried out in the 26 states and the Federal District of Brazil, between February 2021 and September 2023.

### Study population and sampling

The study population consisted of Brazilian women, over 18 years of age, who lived in favelas and were willing to voluntarily participate in the research. The inclusion criteria were as follows: being a woman, 18 years of age or older, living in a favela in Brazil and providing informed consent to participate in the study. Participants who did not self-identify as women and did not complete the questionnaire were excluded from the study.

Regarding the sampling design, the study employed a sequential sampling technique, where participants were included as they were located and agreed to take part in the study [27]. Nevertheless, the calculation for finite populations was used, according to the following parameters: 95% confidence level, random sampling error of 5%, test power of 80% and variance of 50%, since the event is unknown, with an additional 10% referring to possible losse, giving a minimum sample of 385 people.

### Instrument and data collection

The questionnaire used was the “COVID-19 Social Thermometer: Social Opinion” [28, 29]. This questionnaire used was based on an instrument adapted from questions in the National Health Survey of Portugal [28]. Together with the COVID-19 Rapid Quantitative Assessment Tool of WHO [29], validated and published in distinct studies [30–32] of a group of researchers from the National School of Public Health of the New University of Lisbon (ENSP-UNL). For Brazil, the questionnaire was adapted and submitted to a validation process using the Delphi technique. In this validation process, the questionnaire proved to be sufficiently valid and reliable for application in the Brazilian context, including vulnerable populations. All the aspects of the instrument, including its aesthetics and availability, were managed through the RED-Cap software in its offline version.

The “COVID-19 Social Thermometer: Social Opinion” consists of 141 variables, including checklist items, multiple-choice questions, and Likert scale items. The validated version for Brazil is available at the following link: 10.6084/m9.figshare.25917571.

The questionnaire was applied by field interviewers, using cell phones and/or tablets, with an average application time was 20 to 30 min. Interviewers underwent training by the research coordinator to ensure consistent application of the instrument and to prevent measurement bias. To initiate the questionnaire, participants were presented with the Informed Consent Form (ICF) which was read to them. The interview commenced only after participants had agreed and signed the ICF. The ICF was structured in duplicate, which were read in full by the participant and the responsible interviewer. The instrument was applied only once to each person. In the case of illiterate individuals, fingerprints were taken, with each party retaining a copy. The interviewer emphasized the importance of retaining the document. It should be noted that participation in the survey was voluntary.

It is important to highlight that the same instrument has already been used in studies that used the same approach to data collection and have already been published in journals with selective editorial policy, with different objectives such as Rosa et al. [33] as which identified factors associated with self-perception of changes in mental health during the COVID-19 pandemic in Brazil, Moura et al. [34] analyzed the social impact of COVID-19 on international migrants and refugees in terms of loss of income, food insecurity and other social inequalities and Silva [35] analyze the factors associated with agreement with the flexibility of protection measures in Brazil during the COVID-19 pandemic.

### Data analysis

After consistent analysis of the database, descriptive analyses (absolute and relative frequency) were conducted to characterize the profile of the individuals who answered the questionnaire. The data was tabulated in electronic spreadsheets using Microsoft Office Excel 2010 and then imported for analysis in RStudio, version 4.3.2.

### Logistic regression

To verify the social factors associated with violence during the COVID-19 pandemic and the characteristics associated with women who consider their mental health to be poor, two binary logistic regressions were carried out. The first binary logistic regression used the dependent variable “women’s poor self-perception of mental health” and the second binary logistic regression used the dependent variable “women who suffered marital violence (physical violence, psychological violence, sexual violence) during the pandemic”. It is important to note that all the variables and their response categories were dichotomized (0 and 1) to be included in the binary logistic regression model.

For the final model of the binary logistic regressions, a collinearity analysis was carried out between the independent variables using the variance inflation factor (VIF), excluding those with values greater than 10 [36]. The modeling followed the backward stepwise method, starting with all the variables and systematically removing the least relevant ones, based on the lowest value of the Akaike Information Criterion (AIC) [37]. The final model included the calculation of the Odds Ratio (OR) and its 95% Confidence Intervals (95%CI), and was validated using the Cox-Snell, Nagelkerke and McFadden likelihood ratio tests. The predictive capacity was assessed by the area under the ROC curve [38] and its 95%CI, with all the analyses carried out in the RStudio 4.3.2 software.

### Ethical aspects of the research

The project was approved by EERP-USP and ENSP-Fiocruz, respectively under CAAE numbers 32210320.1.3001.5393 and CAAE: 32210320.1.0000.5240. The investigation was conducted in accordance with Resolution No. 466, of December 12, 2012, of the National Health Council, considering the relevant ethical and scientific foundations. Considering the context of the data collection, the researchers adopted strict ethical conduct, ensuring the integrity, respect and safety of the participants. This involved not only obtaining informed consent and keeping the data confidential, but also implementing safety protocols to protect the women involved from any additional risk. These protocols included strategies to minimize the risk of re-victimization and, if necessary, referrals to psychological and legal support were offered. Incorporating these guidelines and good ethical practices strengthens the validity of the study and demonstrates a commitment to the well-being of the participants.

## Results

A total of 766 women living in favelas answered the questionnaire. The predominant race was black/brown (*n* = 581, 75.8%), and those who were single, separated, or widowed accounted for 51.7% (*n* = 396). Additionally, 43.1% had completed high school (*n* = 330). The majority identified as informal workers (*n* = 361, 47.1%), reported not receive any kind of government aid (*n* = 439, 57.3%) and were users of the SUS (*n* = 742, 96.9%). The data is presented in Table 1.

**Table 1:** Sociodemographic characteristics of the women participating in the study, Brazil, 2023 (*n* = 766)

Variables	*n*	%
**Age**		
18 to 29	218	28,5
30 to 59	449	58,6
60 or more	99	12,9
**Race/color**		
White	163	21,3
Black/Brown	581	75,8
Unknown	22	2,9
**Marital status**		
Widowed, separated or single	396	51,7
Married	320	41,8
Unknown	50	6,5
**Religion**		
Yes	642	83,8
No	100	13,1
Unknown	24	3,1
**Employment status**		
Formal employment	187	24,4
Informal employment	361	47,1
Student	68	8,9
Unemployed	150	19,6
**Educational level**		
No study	14	1,8
Primary School	259	33,8
High school	330	43,1
University level	163	21,3
**Monthly income**		
Less than 1 minimum wage	294	38,4
From 1 to 5 minimum wages	376	49,1
From 5 to 10 minimum wages	07	0,9
Above 10 minimum wages	0	0
No income	34	4,4
Unknown	55	7,2
**Number of people living at the residence**		
1 to 3 people	414	54
3 to 5 people	258	33,7
6 to 10 people	90	11,7
Over 10 people	2	0,3
Unknown	2	0,3
**Receives government assistance**		
Yes	325	42,4
No	439	57,3
Unknown	2	0,3
**Do you have any health insurance**		
Yes	83	10,8
No	683	89,2
**You use Unified Health System***(from Portuguese*,* SUS–Sistema Unico de Saúde)*		
Yes	742	96,8
No	22	2,9
Unknown	2	0,3
**You receive a visit from the Community Health Agent**		
Yes	404	52,7
No	360	47
Unknown	2	0,3
**Do you have a health center in your community or neighborhood?**		
Yes	708	92,4
No	58	7,6

The study involved a total of 766 women, of whom 214 (27.93%) reported experiencing conjugal violence during the pandemic. By applying binary logistic regression analysis, factors associated with a greater likelihood of experiencing situations of conjugal violence were identified. These factors included characteristics such as belonging to the black/brown racial group (OR: 1,05; IC95%: 4,55 − 2,53), being in a married marital status (OR: 2,45; IC95%: 1,18 − 5,19), exposure to drug trafficking (OR: 3,23; IC95%: 1,36 − 7,97).

Additionally, those who experienced suicide and attempted suicide were more likely to experience marital violence (OR: 2,89; IC95%: 8,94 − 9,30). However, as a protective factor, police violence (OR: 0,31; IC95%: 0,09 − 0,97), appeared to be less likely to suffer violence (Table 2).

The validation of the model presented in Table 2 showed that the accuracy of the model, as measured by the area under the ROC curve, was 0.95. The likelihood ratio (p = < 0.01), CoxSnell (0.24), Nagelkerke (0.57) and McFadden (0.50) further supported the validity of the model.

**Table 2:** Social factors associated with violence during the COVID-19 pandemic (*n* = 766)

Variables	OR (IC 95%)	*p*-value	AME	SE	VIF
Color/Race: Black/Brown	1,05 (4,55 − 2,53)	0,90	0.0022	0.0188	9.112
Marital status: Married	2,45 (1,18 − 5,19)	< 0,001*	0.0388*	0.0154	1.144
Have you experienced/seen women in situations of conjugal violence (physical violence, psychological violence, sexual violence) in your place of residence, neighborhood or community during quarantine/social isolation?	3,70 (1,48 − 1,11)	< 0,001*	0.1559*	0.0151	1.309
You and/or your family have lived in your place of residence, neighborhood or community during the drug trafficking quarantine/social isolation	3,23 (1,36 − 7,97)	< 0,001*	0.0506*	0.0182	1.809
Have you and/or your family experienced police violence in your place of residence, neighborhood or community during quarantine/social isolation?	0,31 (0,09 − 0,97)	0,05	-0.0505*	0.0253	1.834
You and/or your family lived in your place of residence, neighborhood or community during the quarantine/social isolation suicide/attempted suicide	2,89 (0,89 − 9,30)	0,07	0.0459	0.0249	1.256

AME: Average Marginal Effect; SE: Standard Error; VIF: Variance inflation factor; *: *p* < 0.05

In the second logistic regression, it was found that individuals of black/brown race/color (OR: 1.03; 95% CI: 0.41–2.77), those with informal occupation/employment (OR: 3,26; IC95%: 1,24 − 9,55), and those with complete higher education (OR: 4,47; IC95%: 1,68 − 1,21), were more likely to consider their mental health status poor during the COVID-19 pandemic. In contrast, married women (OR: 0,10; IC95%: 0,02 − 0,33), and students (OR: 0,09; IC95%: 0,00–0,66) were less likely to consider their mental health status poor.

Compared to the pre-pandemic period of COVID-19, some women reported no change in their feelings (OR: 0,15; IC95%: 0,03 − 0,54). Also, as a protective factor, they kept in touch with family and friends, even at a distance, to cope with the pandemic (OR: 0,35; IC95%: 0,11 − 0,94).

Women who reported poor mental health felt sadder, more discouraged, or cried more easily (OR: 3,13; IC95%: 1,09 − 8,93) and more overwhelmed (OR: 4.13; IC95%: 1,43 − 1,19). They were also more likely to start or increase the use of depressants or antidepressants (OR: 5,53; IC95%: 1,60 − 1,78) (Table 3).

To assess the validity of the model presented in Table 3, the accuracy of the model was evaluated using the area of the ROC curve, which showed a value of 0.88. Additionally, the likelihood ratio (p = < 0.01), CoxSnell (0.12), Nagelkerke (0.35) and McFadden (0.31) tests were conducted.

**Table 3:** Characteristics associated with people who consider their state of mental health to be poor (*n* = 766)

Variables	OR (IC 95%)	Valor *p*	AME	SE	VIF
Marital status: married	0,10 (0,02 − 0,33)	< 0,001*	-0.0895	0.0259	1.359
Employment status: Informal	3,26 (1,24 − 9,55)	< 0,001*	0.0476	0.0206	1.616
Employment status: Student	0,09 (0,00–0,66)	< 0,001*	-0.0952	0.0469	1.525
Educational level: Higher School	4,47 (1,68 − 1,21)	< 0,001*	0.0602	0.0200	1.987
Compared to the period before COVID-19, she felt sadder, more discouraged or cried more easily	3,13 (1,09 − 8,93)	<0,001*	0.0459	0.0211	1.324
Compared to the period before COVID-19, she felt more overwhelmed	4,13 (1,43 − 1,19)	<0,001*	0.0570	0.0213	1.345
Compared to the period before COVID-19, she had no change in her feelings	0,15 (0,03 − 0,54)	< 0,001*	-0.0744	0.0283	2.506
During the pandemic, in order to cope with the situation, I kept in touch with family and friends, even at a distance	0,35 (0,11 − 0,94)	< 0,001*	-0.0417	0.0212	1.276
Women who started or increased their use of tranquilizers or antidepressants during the pandemic	5,53 (1,60 − 1,78)	< 0,001*	0.0687	0.0244	1.101

AME: Average Marginal Effect; SE: Standard Error; VIF: Variance inflation factor; *: *p* < 0.05

## Discussion

This study aimed to scrutinize the frequency of violence against women and their mental health status within favelas during the COVID-19 pandemic in Brazil, exploring how the pandemic may have impacted these issues and identifying factors that contribute to these experiences. Out of the 766 participants interviewed that resides in favelas, 214 (27.93%) reported having experienced conjugal violence during the pandemic period.

The pandemic has created an unexpected scenario, not only in the epidemic-biological field but also in social terms, challenging our understanding of its consequences in various areas of life. Economic, cultural, historical, and political impacts have not been equal for the various social groups, including women.

Gender inequalities, along with factors such as race, territory, regionality, income and schooling, have positioned poor women at the forefront of the groups most affected by the pandemic. The Brazilian state’s abandonment and neglect of women, coupled with the virus epidemic, have resulted in new forms of precarious life and non-recognition of rights [39].

The women participated in our study were primarily aged between 30 and 59 (58.6%). The majority identified themselves as black/brown (75.8%), and were single, widowed or separated (51.7%). About 43.1% had completed high school, while 47.1% had informal employment. The family’s monthly income ranged between 1 and 5 salaries for 49.1% of the participants. Additionally, 83.8% reporting having a religion, 57.3% did not receive emergency aid, 89.2% had no medical insurance and 96.9% used the SUS.

Similarly, a study carried out in Italy [40] showed a predominantly single sample (67.4%), with most completed high school and 15.8% working as freelance professionals. Leoncini [41] highlighted the reality of female-headed households in Brazil, which increased by 105% between 2001 and 2015.

Regarding religiosity, 83.8% of the women interviewed in our study reported having a religion. In recent decades, religiosity/spirituality has gained prominence as a dimension that interferes with the health conditions of the population and is therefore an important social determinant to be considered in public health [42, 43]. It is extremely important to reflect on how religious and spiritual assistance support in the context of health practices can be a positive resource in dealing with complex situations that have arisen or been enhanced by the pandemic. It is extremely important to reflect on how religious and spiritual assistance support in the context of health practices can be a positive resource in dealing with complex situations that have arisen or been enhanced by the pandemic, while also addressing the limitations that such support may have in offering comprehensive care.

The social inequalities prevalent in our country have become more evident with the pandemic, in which around 71.5% of Brazilians did not have any supplementary health service [44], corroborating our study, showing that 89.2% of women did not have a medical insurance and 96.9% were SUS users.

In April 2020, Law No. 13,982/2020 [45] was enacted, which established exceptional social protection measures due to the COVID-19 pandemic and stated that the granting of the benefit was a fundamental right necessary for groups in situations of vulnerability to have their dignity ensured. However, our study found that 57.3% of women living in favelas and/or communities did not receive the aid, which further increases the suffering of these women with the worry of daily expenses. The social protection actions instituted by the Brazilian federal entities have faced problems in the execution and implementation of Emergency Aid, including the regularization of the individual registration (CPF- Cadastro de Pessoa Física) of those applying for the aid [46]. The difficulty of how to proceed with the regularization of the CPF has led women in situations of vulnerability to have difficulty accessing the aid.

In the international literature, research highlights that gender inequalities do not affect all women equally. Black women, particularly in contexts of poverty, are often more vulnerable due to the combined effects of racial and gender-based discrimination. This is evident in studies that examine the disproportionate representation of women of color in extreme poverty, especially in contexts such as Sub-Saharan Africa and Central Asia​ [47]. Gender inequality is strongly linked to women’s economic vulnerability, particularly for those outside the formal labor market or in underemployment. Women’s lower participation in paid work, compared to men, exacerbates poverty. This is often intensified for women in extreme poverty, where limited access to financial resources, education, and opportunities for decent work further entrenches their economic disadvantage​ [47]. A significant factor contributing to women’s poverty is the unequal burden of unpaid care work, which women are more likely to bear, limiting their opportunities for paid employment and economic independence. In many societies, this care work is undervalued and remains unrecognized, contributing to greater poverty among women​ [48]. For women who do not conform to traditional gender norms (such as transgender women and gender non-conforming individuals), there are additional barriers. These include systemic discrimination and violence, which can exacerbate their vulnerability in already marginalized groups, such as those in extreme poverty or underemployment​ [48].

It is known that black and brown people are more prone to situations of social vulnerability due to the structural inequalities of Brazilian society [49]. In addition, this population is susceptible to other risks, as pointed out in a study [50] in which the average number of deaths from COVID-19 in the five macro-regions of Brazil was higher among black people, which shows the high degree of exposure of this population to COVID-19, an aspect that is related to various social and structural difficulties.

The data found in this study corroborates the data found in the literature, in which the increase in violence against women during the period of social distancing has been observed in different countries, such as China, the United Kingdom, the United States, France and Brazil [51–56].

During the COVID-19 pandemic, the context of social isolation and economic tensions has exacerbated property and moral violence against women. In addition to physical and sexual violence, other forms of aggression - including property violence, characterized by the control or destruction of the victim’s assets, and moral violence, with humiliation and attacks on dignity - have intensified, especially in domestic environments. These abuses emerge from relationships of power and control intensified by the restrictions of the period and by the often accentuated financial dependence, severely impacting the mental health and well-being of the women affected [57].

In China, police records of domestic violence have tripled during the epidemic [58]. In Italy [59], France [60] and Spain [61] an increase in the occurrence of domestic violence was also observed after the implementation of mandatory home quarantine. Married women were more likely to suffer violence from their intimate partners, corroborating other studies carried out in Thailand [62] and Uganda [63], which found that 61.1% and 55.4% of women who suffered violence during the pandemic were married, respectively.

In relation to the greater likelihood of women who have experienced drug trafficking OR: 3,23; IC95%: 1,36 − 7,97), suffering violence, according to UN Women data, there has been an increase in violence from drug trafficking, has left these women more vulnerable to suffering violence [64]. During the lockdown, drug trafficking intensified in the large favelas, claiming many victims, and leaving children and adolescents orphaned without recognition of their rights.

Police violence appears to be a protective factor against marital violence. Considering that the police are often the woman’s first point of contact and are also the ones who will lead the aggressor away, since their purpose is to curb domestic violence, to always be attentive to the victim’s vulnerability, in all its different facets, in order to prevent revictimization or even institutional violence against this woman who seeks her last or only recourse in the state [65].

Several studies in Brazil contradict the view that exposure to police violence could protect women from domestic violence, suggesting that institutional violence generally exacerbates women’s vulnerability, especially in peripheral contexts. According to the Brazilian Public Security Forum [66], the presence of police violence is often associated with increased tensions within favelas, negatively affecting women’s psychological and social well-being and potentially increasing the risk of domestic violence. These contexts of constant institutional aggression aggravate situations of stress and domestic conflict, especially in areas of high social vulnerability. In addition, Atlas of Violence [67] highlights that, rather than protecting, exposure to police violence can make women in favelas more likely to suffer multiple forms of violence, including domestic violence, through the normalization of violence and the fear of seeking help from public institutions seen as hostile or ineffective. These analyses suggest that, rather than offering protection, police violence contributes to an environment of generalized insecurity, reducing support and resources for women facing domestic violence.

Still on the impacts of the COVID-19 pandemic, it has possibly promoted a confluence of risk factors for suicide, such as a significant increase in mental disorders (for example, depression and post-traumatic stress disorder), leading to an increase in suicide rates and suicide attempts in the longer term [68]. In Brazil, a study carried out by the Oswaldo Cruz Foundation [69] evaluated suicides in Brazil in 2020, during the pandemic, and identified an overall drop of 13% in suicide rates in the general Brazilian population, however, a 40% increase in suicides was observed among women aged 60 and over in the Northeast region, this could reveal some aspects of socioeconomic inequality across regions and gender in the country.

Faced with all these situations, the women who reported having their mental health damaged had a higher prevalence of situations such as being more agitated, anxious, sad, discouraged and crying more easily. They were also more likely to feel overwhelmed.

The results found in this study which the pandemic has been as a negative factor for women’s mental health, negatively affecting all aspects of life and leaving them more vulnerable to developing mental disorders. These studies [70–73] show that the pandemic has caused mental and physical damage, as well as lead to moderate and severe psychological conditions, such as post-traumatic stress disorder, depression, anxiety, panic disorder and behavioral disorders.

Being married and being a student appeared as a protective factor for better mental health, and one study revealed that psychological distress during the pandemic also affected those who lived alone and had no children [74]. However, the literature points to students as one of the groups most affected psychologically during the pandemic. A study that assessed 460 Portuguese university students (with an average age of 20.14 years) using the DASS-21 observed high levels of anxiety, depression and stress in this population [75]. In Spain, 2,530 students and workers at the University of Valladolid showed moderate to extremely severe levels of symptoms of anxiety (21.34%), depression (34.19%) and stress (28.14%) [76]. A study examined the impact of the pandemic on Brazilian university students, focusing on anxiety, obsessive-compulsive behaviors and emotional well-being. The research found that 37% of participants reported high levels of anxiety and 46.1% experienced moderate levels of obsessive-compulsive thoughts and behaviors. The study also highlighted the negative correlation between positive emotions and anxiety, as well as compulsive behaviors [77].

On the other hand, having completed higher education has been identified as a factor in poor mental health, and a study carried out in Germany [74] found that 40.6% of women who had completed higher education had mental disorders and had started or increased dangerous substance use due to the COVID-19 pandemic, which alerts us in terms of public health, since both entail high individual and social burdens.

The pandemic has also caused an economic crisis that has hit many citizens with unemployment and loss of income, and certainly those in situations of social vulnerability have been the hardest hit. Around 40% of citizens do informal work in Brazil [78] and do not have the support of labor rights or other sources of income [79].

A study carried out in the Federal District of Brazil [80] investigated the average monthly consumption of antidepressants. There was an increase of 181.90%, 124.36%, 325.33% and 125% in the use of Fluoxetine, Amitriptyline, Imipramine and Clomipramine respectively. With regard to anxiolytics, there was an increase in consumption for both Diazepam, Clonazepam and injectable Clonazepam, of 12.80%, 22.18% and 6.45% in that order. The results also indicate that women consume more antidepressants and anxiolytics. The double workload of this population, also taking care of the family and the home, together with the accumulation of professional activities, as a result of the greater inclusion of women in the labor market, may have contributed to increasing mental health demands in women [81, 82].

Added to these factors is the fact that during the pandemic, women who had already been diagnosed with depression and anxiety, who need longitudinal follow-up, were possibly left without this assistance, since health care services were overloaded with COVID-19.

The women reported maintaining contact with their families, even at a distance, as a way of alleviating the problems triggered by the pandemic. Contact with supportive social ties, including family, friends or healthcare providers [83], protects against depression by buffering the effects of stress and improving coping skills [84].

A survey carried out in Oregon found that before the pandemic, the average time spent interacting socially in person, by phone/video call and by text/email/letter was 406, 141 and 68 min/week respectively. However, during isolation, the time spent in person was reduced by 135 min/week, while the time spent on the phone/video call and writing increased by 33 and 26 min/week respectively. An increase of one hour of writing per week, enabling interaction via text/email/letter with friends during the pandemic, was associated with a 23% decrease in the likelihood of feeling low mood [85].

### Limitations

The study’s limitations include the study design employed. As with all non-probabilistic sampling, it is not “representative” and generalizations cannot be made about a population, and the study participants may not be very diverse. It is therefore important that more studies are carried out to understand the real impact of the COVID-19 pandemic in relation to violence and the mental health of Brazilian women living in favelas. The analysis of self-reported conditions rather than formal mental health diagnoses is another limitation to be considered in this study.

Underreporting may also be a limitation, since violence against women is a sensitive issue, often shrouded in stigma, fear of reprisals and feelings of shame or guilt. This underreporting can occur both because participants are reluctant to reveal their experiences, especially in contexts where there is little support network or where the aggressor is someone close to them, and because they are afraid of reliving traumas during the interview process. Furthermore, even with the guarantee of anonymity and confidentiality, some women may fear exposure and social judgment, which leads them to omit details or even refuse to participate. This limitation must be taken into account when interpreting the data, as it may result in an underestimation of the real prevalence and different manifestations of the violence experienced.

### Theoretical and practical implications

This study expands knowledge about gender-based violence in contexts of social vulnerability by highlighting how the COVID-19 pandemic has intensified marital violence and mental health problems among women living in favelas in Brazil. The research highlights specific factors that increase the risk of violence and mental distress, including race, marital status, involvement in drug trafficking and informal employment conditions. These findings provide a deeper insight into the multiple dimensions of vulnerability, demonstrating how the combination of social inequalities and crisis contexts exacerbate gender-based violence and psychological distress in marginalized communities.

The study’s intersectional approach makes it possible to understand how different layers of inequality, such as race, economic status and marital status, influence experiences of violence and mental health. By integrating these variables, the research offers a more complex view of vulnerability and a solid basis for future investigations to explore the intersections of these factors more broadly. Thus, the study opens up avenues for research that seeks to identify how the overlapping of unfavorable conditions uniquely affects the lives of women in favelas, offering a robust theoretical framework for analyzing the relationship between inequalities and vulnerabilities.

In addition, the study contributes to the theory that health crises, such as the COVID-19 pandemic, act as amplifiers of existing vulnerabilities in disadvantaged social contexts. The pandemic has opened up and intensified pre-existing inequalities, especially in slums, highlighting the urgency of studies that assess the disproportionate impact of global crises on vulnerable social groups. These findings reinforce the importance of including gender and race perspectives in formulating responses to public health crises, in order to protect the most susceptible populations and mitigate the adverse effects of future pandemics.

## Conclusions

The pandemic has represented a health crisis that has especially exacerbated the social vulnerability of these poor women from favelas and communities, living in precarious living conditions due to their social determinants of health such as gender, race, unemployment and territory. During the pandemic, the difficulties faced by these anonymous, black, peripheral and poor women, who depend on public services, became even more evident, leaving deep marks on their trajectories.

It is worth noting that Gender Equality and Reducing Inequalities are part of the global targets of the Sustainable Development Goals, seeking to reduce violations against women and to reach all women who still lack access to their rights and to live without violence. These inequalities are deeply rooted in the socio-historical and cultural construction of our country, which places these women as one of the groups most affected during the pandemic, and thus justifies the importance of mainstreaming gender in the formulation of public policies in times of pandemic.

## Electronic supplementary material

Below is the link to the electronic supplementary material.

Supplementary Material 1

Supplementary Material 2

## Data Availability

The datasets used and/or analysed during the current study available from the corresponding author on reasonable request.

## Abbreviations

AIC: Akaike Information Criterion
AME: Average Marginal Effect
COVID-19: Coronavírus Disease 2019
CPF: Individual Registration (Cadastro de Pessoa Física)
EERP-USP: Ribeirão Preto College of Nursing at University of São Paulo
ENSP-Fiocruz: National School of Public Health at the Osvaldo Cruz Foundation
ENSP-UNL: National School of Public Health at the New University of Lisbon
IBGE: Brazilian Institute of Geography and Statistics (Instituto Brasileiro de Geografia e Estatística.)
ICF: Informed Consent Form
IPV: Intimate partner violence
OR: Odds Ratio
ROC curve: Receiver Operating Characteristic curve
SE: Standard Error
SUS: Unified Health System (Sistema Unico de Saúde)
VIF: Variance Inflation Factor
WHO: World Health Organization
95% CI: 95% Confidence Intervals
