# Supplementary material for: Violence experienced by women, their mental health status, and determinants in favelas under the pandemic COVID-19 in Brazil
Source: BMC Womens Health. 2025 May 21;25:242. doi: 10.1186/s12905-025-03793-1 (PMC12093629; doi:10.1186/s12905-025-03793-1)
Supplement: Supplementary file 2 — Supplementary Material 2 [file 12905_2025_3793_MOESM2_ESM.pdf]

PARECER CONSUBSTANCIADO DO CEP

DADOS DO PROJETO DE PESQUISA

Título da Pesquisa: TERMÔMETRO SOCIAL COVID-19: Fatores associados à percepção de risco, aos padrões de comportamento e à adesão às medidas de proteção que influenciam no combate à pandemia no Brasil

Pesquisador: Ricardo Alexandre Arcêncio

Área Temática:

Versão: 2

CAAE: 57933622.4.1001.5393

Instituição Proponente: Escola de Enfermagem de Ribeirão Preto - USP

Patrocinador Principal: Financiamento Próprio

DADOS DO PARECER

Número do Parecer: 5.512.199

Apresentação do Projeto:

Trata-se de respostas a pendências apresentadas por este CEP em Parecer Consubstanciado: 5.409.514, de 16 de maio de 2022.

Objetivo da Pesquisa:

Objetivo Primário:

Analisar os fatores associados à percepção de risco, aos padrões de comportamento e à adesão às medidas de proteção que influenciam no combate a COVID 19 no Brasil.

Objetivo Secundário:

1. Caracterizar os participantes segundo as suas características sóciodemográficas e de vulnerabilidade;
2. Levantar percepções e comportamentos de risco ao longo das mudanças marcantes no padrão evolutivo da pandemia;
3. Identificar fatores epidemiológicos, sócio-políticos e culturais associados a estas percepções de risco e comportamentos;
4. Verificar a adesão às medidas de proteção adotadas pela população geral e populações em situação de vulnerabilidade social, para mitigar os riscos de contaminação pela COVID-19, fatores associados e ainda seus desafios;

5. Estabelecer um diálogo com a sociedade organizada (movimentos sociais, organizações comunitárias) atuantes nos territórios em situação de vulnerabilidade social onde se realizará a pesquisa, tendo em vista a realização de diagnósticos e o desenvolvimento de estratégias conjuntas, que contribuam para evidenciar e alterar positivamente a percepção e o comportamento de risco em relação a COVID-19;
6. Avaliar a adesão à vacina e ainda os fatores e ou explicações quando os participantes neguem o uso dessa medida de proteção;
7. Verificar o comportamento de risco coletivo e ou medidas de proteção ou prevenção coletiva, expressos respectivamente pelo distanciamento social e adesão à cobertura da vacina contra a COVID-19, ao longo do ciclo evolutivo da pandemia na população geral e na população em situação de vulnerabilidade social;
8. Identificar territórios ou municípios em risco pela baixa adesão ao distanciamento social e ou baixa cobertura da vacina contra COVID-19, e suas consequências, expressas por seus indicadores de morbimortalidade, na população geral e nas populações em situação de vulnerabilidade social;
9. Adotar estratégias para a translação do conhecimento para a política pública e aos serviços de saúde.

Avaliação dos Riscos e Benefícios:

Tópico já apreciado.

Comentários e Considerações sobre a Pesquisa:

Vide tópico "Conclusões ou Pendências e Lista de Inadequações".

Considerações sobre os Termos de apresentação obrigatória:

Vide tópico "Conclusões ou Pendências e Lista de Inadequações".

Recomendações:

Vide tópico "Considerações Finais a Critério do CEP".

Conclusões ou Pendências e Lista de Inadequações:

1. Sobre o documento PROJETO_DETALHADO.pdf: solicita-se que o pesquisador encaminhe projeto detalhado com informações sobre as atividades que serão desenvolvidas neste centro de pesquisa (EERP/USP). Essas informações devem constar também na Plataforma Brasil.

RESPOSTA DO PESQUISADOR:

Frente ao aspecto apresentado acima, foi alterado o documento "PROJETO_DETALHADO.pdf". Incluiu-se o tópico "5.2. Breve contexto cronológico para acompanhamento da Fases do projeto", que faz alusão as atividades que foram desenvolvidas pela ENSP-FIOCRUZ e aquelas que seguirão sob coordenação pelo centro de pesquisa da EERP/USP.

PARECERISTA: PENDÊNCIA ATENDIDA.

2. Sobre os documentos TCLE_OFF.pdf e TCLE_ON.pdf: devem ser incluídos os dados completos para contato com este CEP, bem como informações para contato com o pesquisador responsável (e-mail e telefone). Sugestão para os dados deste CEP: Este projeto foi aprovado pelo Comitê de Ética em Pesquisa da Escola de Enfermagem de Ribeirão Preto – USP (CEP-EERP/USP), que tem a finalidade de proteger eticamente o participante de pesquisa, sob o número do CAAE:______. Caso você tenha alguma dúvida sobre a ética da pesquisa, entre em contato com o CEP-EERP/USP pelo telefone (16) 3315-9197, e-mail: cep@eerp.usp.br ou no endereço: Av. dos Bandeirantes, 3900- Ribeirão Preto, SP. Horário de atendimento: de segunda a sexta-feira, em dias úteis, das 10 às 12 e das 14 às 16 horas.

RESPOSTA DO PESQUISADOR:

Frente ao aspecto apresentado acima, foi alterado o item TCLE_OFF.pdf e TCLE_ON.pdf. Incluiu-se o texto conforme sugestão, segue: "Este projeto foi aprovado pelo Comitê de Ética em Pesquisa da Escola de Enfermagem de Ribeirão PretoUSP (CEP-EERP/USP), que tem a finalidade de proteger eticamente o participante de pesquisa, sob o número do CAAE: 57933622.4.1001.5393. Caso você tenha alguma dúvida sobre a ética da pesquisa, entre em contato com o CEP-EERP/USP pelo telefone (16) 3315- 9197, e-mail: cep@eerp.usp.br ou no endereço: Avenida dos Bandeirantes, 3900 – Ribeirão Preto, SP. Horário de atendimento: de segunda a sexta-feira, em dias úteis, das 10 às 12 e das 14 às 16 horas.

PARECERISTA: PENDÊNCIA ATENDIDA.

Considerações Finais a critério do CEP:

Tendo em vista trata-se de etapas que serão realizadas pelo pesquisador responsável na EERP/USP e que o pesquisador faz parte da equipe do projeto multicêntrico já aprovado por este CEO, o CEP-EERP/USP considera que o protocolo de pesquisa ora apresentado contempla os quesitos éticos necessários, estando apto a ser iniciado a partir da presente data de emissão deste parecer.

Em atendimento ao subitem II.19 da Resolução CNS 466/2012, cabe ao pesquisador responsável pelo presente estudo elaborar e apresentar relatórios parcial e final "[...] após o encerramento da pesquisa, totalizando seus resultados", em forma de "notificação". O modelo de relatório do CEP-EERP/USP se encontra disponível em:
http://www.eerp.usp.br/research-comite-etica-pesquisa-relatorio/

Parecer apreciado ad referendum.

Este parecer foi elaborado baseado nos documentos abaixo relacionados:

Tipo Documento	Arquivo	Postagem	Autor	Situação
Informações Básicas do Projeto	PB_INFORMAÇÕES_BÁSICAS_DO_PROJETO_1909507.pdf	02/06/2022 20:29:03		Aceito
Outros	Of_resposta_parecer_CEP.pdf	02/06/2022 19:59:26	Ricardo Alexandre Arcêncio	Aceito
Cronograma	CRONOGRAMA.pdf	02/06/2022 19:57:57	Ricardo Alexandre Arcêncio	Aceito
Projeto Detalhado / Brochura Investigador	PROJETO_DETALHADO.pdf	02/06/2022 19:57:45	Ricardo Alexandre Arcêncio	Aceito
TCLE / Termos de Assentimento / Justificativa de Ausência	TCLE_ON.pdf	02/06/2022 19:56:15	Ricardo Alexandre Arcêncio	Aceito
TCLE / Termos de Assentimento / Justificativa de Ausência	TCLE_OFF.pdf	02/06/2022 19:55:58	Ricardo Alexandre Arcêncio	Aceito
Outros	OFICIO_DE_ENCAMINHAMENTO_DE_PROJETO_AO_CEP.pdf	18/04/2022 01:03:24	Ricardo Alexandre Arcêncio	Aceito
Outros	TERMO_GARANTIA_RETORNO_BENEFICIOS_PARTICIPANTES_PESQUISA.	12/04/2022 15:44:15	Ricardo Alexandre Arcêncio	Aceito
Folha de Rosto	FOLHA_DE_ROSTO.pdf	12/04/2022 15:36:15	Ricardo Alexandre Arcêncio	Aceito

Endereço: BANDEIRANTES 3900
Bairro: VILA MONTE ALEGRE **CEP:** 14.040-902
UF: SP **Município:** RIBEIRAO PRETO
Telefone: (16)3315-9197 **E-mail:** cep@eerp.usp.br

Outros	MINUTA_DE_CONVENIO.pdf	12/04/2022 15:35:49	Ricardo Alexandre Arcêncio	Aceito
Outros	INSTRUMENTO_DE_PESQUISA.pdf	12/04/2022 15:34:16	Ricardo Alexandre Arcêncio	Aceito
Outros	DECLARACAO_DE_COMPROMISSO_DO_PESQUISADOR_RESPONSAVEL.	12/04/2022 15:33:36	Ricardo Alexandre Arcêncio	Aceito
Parecer Anterior	APROVACAO_CEP_FIOCRUZ.pdf	12/04/2022 15:32:11	Ricardo Alexandre Arcêncio	Aceito
Parecer Anterior	APROVACAO_CEP_EERP_USP.pdf	12/04/2022 15:31:47	Ricardo Alexandre Arcêncio	Aceito
Declaração do Patrocinador	DECLARACAO_DO_PATROCINADOR.pdf	12/04/2022 15:29:11	Ricardo Alexandre Arcêncio	Aceito
Orçamento	ORCAMENTO.pdf	12/04/2022 15:27:05	Ricardo Alexandre Arcêncio	Aceito
Declaração de Instituição e Infraestrutura	DECLARACAO_DA_EXISTENCIA_DE_INFRAESTRUTURA.pdf	12/04/2022 15:24:00	Ricardo Alexandre Arcêncio	Aceito

Situação do Parecer:
Aprovado

Necessita Apreciação da CONEP:
Não

RIBEIRAO PRETO, 06 de Julho de 2022

Assinado por:
RONILDO ALVES DOS SANTOS
(Coordenador(a))

Endereço: BANDEIRANTES 3900
Bairro: VILA MONTE ALEGRE **CEP:** 14.040-902
UF: SP **Município:** RIBEIRAO PRETO
Telefone: (16)3315-9197 **E-mail:** cep@eerp.usp.br
